# Effects of Exposure to Ozone on the Ocular Surface in an Experimental Model of Allergic Conjunctivitis

**DOI:** 10.1371/journal.pone.0169209

**Published:** 2017-01-03

**Authors:** Hun Lee, Eung Kweon Kim, Hee Young Kim, Tae-im Kim

**Affiliations:** 1 Department of Ophthalmology, International St. Mary's Hospital, Catholic Kwandong University College of Medicine, Incheon, South Korea; 2 Department of Ophthalmology, The Institute of Vision Research, Yonsei University College of Medicine, Seoul, South Korea; 3 Department of Ophthalmology, Corneal Dystrophy Research Institute, Yonsei University College of Medicine, Seoul, South Korea; University of Kansas Medical Center, UNITED STATES

## Abstract

Based on previous findings that ozone can induce an inflammatory response in the ocular surface of an animal model and in cultured human conjunctival epithelial cells, we investigated whether exposure to ozone exacerbates symptoms of allergic conjunctivitis. We evaluated the effects of exposure to ozone on conjunctival chemosis, conjunctival injection, corneal and conjunctival fluorescein staining scores, production of inflammatory cytokines in tears, and aqueous tear production in a mouse model of allergic conjunctivitis. To validate our *in vivo* results, we used interleukin (IL)-1α-pretreated conjunctival epithelial cells as an *in vitro* substitute for the mouse model. We evaluated whether exposure to ozone increased the inflammatory response and altered oxidative status and mitochondrial function in IL-1α-pretreated conjunctival epithelial cells. In the *in vivo* study, ozone induced increases in conjunctival chemosis, conjunctival injection, corneal and conjunctival fluorescein staining scores, and production of inflammatory cytokines, accompanied by a decrease in tear volume. In the *in vitro* study, exposure to ozone led to additional increases in IL-6 and tumor necrosis factor-α mRNA levels, which were already induced by treatment with IL-1α. Ozone did not induce any changes in cell viability. Pretreatment with IL-1α increased the expression of manganese superoxide dismutase, and exposure to ozone led to additional increments in the expression of this antioxidant enzyme. Ozone did not induce any changes in mitochondrial activity or expression of mitochondrial enzymes and proteins related to mitochondrial function, with the exception of phosphor-mammalian target of rapamycin. Treatment with butylated hydroxyanisole, a free radical scavenger, attenuated the ozone-induced increases in IL-6 expression in IL-1α-pretreated conjunctival epithelial cells. Therefore, we conclude that exposure to ozone exacerbates the detrimental effects on the integrity of the ocular surface caused by conjunctival allergic reactions, and further increases the inflammatory response in IL-1α-pretreated conjunctival epithelial cells.

## Introduction

Ozone, produced by reactions between nitrogen oxides and volatile organic compounds in a process catalyzed by ultraviolet light, is regarded as one of the most toxic air pollutants to which humans are routinely exposed.[1] The respiratory tract, cutaneous tissue, and exposed ocular tissue are expected to be affected by atmospheric exposure to ozone directly or indirectly.[2–4] Exposure to high concentrations of ozone has been reported to cause damage to the ocular surface, breakdown of the integrity of the corneal epithelium, and increased inflammatory tear cytokine levels in a mouse model.[3]

The toxicity of tropospheric ozone is presumed to be caused by ozone-mediated oxidative damage to various biomolecules.[5] Ozone can be rapidly converted into a number of reactive oxygen species (ROS) and exerts its toxic effects by reacting with cell proteins and lipids.[6] Overproduction of ROS results in oxidative stress, which can be an important mediator of damage to lipids, membranes, proteins, and DNA, and can lead to apoptosis or necrosis depending on the severity of the oxidative stress.[7] However, the beneficial effects of ROS, which are demonstrated in the defense against infectious agents, in the function of a number of cellular signaling pathways, and in the induction of mitogenic responses, are associated with low to moderate concentrations of ozone.[8–10]

According to previous studies evaluating the effects of ozone on the respiratory tract, exposure to ozone has been reported to decrease pulmonary function, increase airway responsiveness, and induce airway inflammation in humans and experimental animal models.[11–15] In the rat alveolar macrophage, exposure to ozone increases the expression of interleukin (IL)-1α and IL-1β and the secretion of pro-inflammatory cytokines.[16] Further, individuals with allergic asthma are prone to exacerbation of allergic inflammation because of their primed inflammatory state, along with elevated sputum levels of IL-1β and neutrophilia before and after exposure to ozone.[17–19] In addition, individuals with allergic asthma have demonstrated evidence of an increased innate immune response to exposure to ozone.[17,18,20]

Allergic conjunctivitis, a common ocular immune disorder, is mediated by pathways similar to those occurring in other allergic diseases, including asthma.[21] The immunopathogenic mechanisms in allergic conjunctivitis involve reactions mediated by immunoglobulin E (IgE) or T-helper 2 cells.[22] The IgE-mediated conjunctival allergic reaction induced by specific conjunctival provocation results in infiltration of predominantly mast cells and eosinophils that produce various inflammatory cytokines.[23,24]

Based on the impact of exposure to ozone on aggravation of allergic asthma, we hypothesized that exposure to ozone would similarly exacerbate the symptoms of allergic conjunctivitis. Conjunctival epithelium directly exposed to external insults can be simultaneously affected by ambient ozone and allergens. To confirm our hypothesis, we investigated the effects of exposure to ozone on the ocular surface in a mouse model of experimental allergic conjunctivitis (EAC). To confirm our *in vivo* results, we used a separate *in vitro* model consisting of IL-1α-pretreated conjunctival epithelial cells, which we found to show an inflammatory response similar to that in allergic conjunctivitis. We evaluated whether exposure to ozone increased the inflammatory response and altered oxidative status and mitochondrial function in these cells. We also studied the effects of butylated hydroxyanisole (BHA), a free radical scavenger, on the expression of IL-6, an inflammatory cytokine, in response to exposure to ozone in these cells.

## Materials and Methods

### Animal

This study was conducted in strict accordance with and adherence to the relevant national and international guidelines regarding animal handling as mandated by the Institutional Animal Care and Use Committee (IACUC) of the Yonsei University Health System (Seoul, Korea). The committee reviewed and approved the animal study protocol (approval number 2014–0107). All experimental protocols were conducted in accordance with the tenets of the Declaration of Helsinki and the Association for Research in Vision and Ophthalmology (ARVO) Statement on the Use of Animals in Ophthalmic and Vision Research.

Forty-six female BALB/C mice (Orient Bio, Seongnam, Korea), aged 6–8 weeks, were housed under a 12-hour light/dark schedule (lights on at 6 am, off at 6 pm) with access to autoclaved food and water *ad libitum*, and were treated humanely with regard to minimization of suffering.[25] All mice were maintained in an experimental animal facility under specific pathogen-free conditions.

### Experimental allergic conjunctivitis and exposure to ozone

The mice were randomly divided into 4 groups: group A, negative control (n = 10); group B, EAC with exposure to filtered room air (n = 10); group C, EAC with exposure to 0.5 parts per million (ppm) of ozone (n = 10); and group D, EAC with exposure to 2.0 ppm of ozone (n = 10). To generate EAC in the right eye before exposure to ozone, the mice were sensitized intraperitoneally with 1 μg of ovalbumin (OVA, Grade V; Sigma-Aldrich, St. Louis, MO, USA) and 200 μL of 1.5% aluminum hydroxide (ALUM; Pierce, Rockford, IL, USA) on days 0 and 7, and then challenged topically in the conjunctival sac of the right eye with 250 μg of OVA on days 15 and 18.[26,27]

As described in detail previously, [3] the mice were exposed to filtered room air (group B), 0.5 ppm of ozone (group C), or 2.0 ppm of ozone (group D) for 2 hours in a whole-body exposure chamber consisting of a Teflon-lined clear acrylic box (50 × 50 × 50 cm) with three holes, i.e., an ozone gas inlet, an ozone gas outlet, and a hole for monitoring ozone concentration. The exposure was routinely performed within the period from 7 pm to 10 pm to ensure that the mice were in an awake active state. The exposure was repeated every day for 2 weeks, starting from day 19. The mice were placed in the same position, such that their right flank was exposed to the inlet of the chamber. Ozone was generated with an OA-2 ozone generator (Ozone Engineering, Seoul, Korea). The concentration of ozone within the chamber was continuously monitored using a PortaSens II gas detector (Analytical Technology Inc., Collegeville, PA, USA). Temperature (20°C–22°C) and humidity (50%–60%) were maintained at a constant level within the chamber.

### Evaluation of clinical findings in experimental allergic conjunctivitis

The mice were anesthetized using a mixture of Zoletil (30 mg/kg, intraperitoneal injection; Virbac Laboratories, Carros, France) and Rompun (10 mg/kg, intraperitoneal injection; Bayer Korea, Seoul, Korea). The corneas were routinely kept moist with regular application of preservative-free 0.5% carboxymethylcellulose (Refresh Plus; Allergan Inc., Irvine, CA, USA). Conjunctival chemosis, conjunctival injection, and corneal and conjunctival fluorescein staining scores, all of which are parameters representative of ocular surface inflammation, were measured by an observer blinded to the experimental groups before exposure to ozone (baseline), and after 1 and 2 weeks of exposure. Conjunctival chemosis and injection were graded from 0 to 4 according to severity (0; no finding, 1; mild, 2; moderate, 3; severe, 4; very severe). Fluorescein staining was performed by applying 5% fluorescein (0.5 μL) into the inferior conjunctival sac using a micropipette. The cornea and conjunctiva were examined using a slit lamp with maximum cobalt blue light 3 minutes after instillation of fluorescein. The eyes were closed between evaluations to prevent excessive exposure and irritation of the ocular surface. The corneal surface was divided into five areas, and punctate staining in each area was scored from 0 to 3 in a blinded fashion using a standardized (National Eye Institute) grading system.[28] The conjunctival punctate staining in each nasal and temporal area was scored from 0 to 5 in a blinded fashion using a standardized grading system (Oxford staining score). The final reported score was an average of three separate measurements. Representative photographs were taken using a single-lens reflex camera (Canon, Tokyo, Japan) connected to a slit lamp.

For analysis of the cytokines in tears, which indirectly reflect inflammation of the ocular surface, before exposure to ozone (baseline), and after 1 and 2 weeks of exposure, 1.5 μL of phosphate-buffered saline (PBS; Millipore, Billerica, MA, USA) was injected into the inferior conjunctival sac using a micropipette. Approximately 1 μL of tear fluid in buffer was collected using a micropipette. Irritation of the ocular surface and lid margin was minimized by collecting unstimulated tear fluid from the marginal tear strip of the lower lid near the lateral canthus. Tear samples were immediately transferred to 0.5 mL Eppendorf tubes (Eppendorf, Fremont, CA, USA), placed on dry ice, and kept in a -70°C freezer until use for immunoassay. The cytokines (IL-1β, IL-6, IL-17, and tumor necrosis factor [TNF]-α) were measured by Luminex technology with a Milliplex Analyzer Luminex 200 System (MCYTOMAG-70K-08 Mouse Cytokine Magnetic Kit; Merck Millipore).

Aqueous tear production, one of the most important parameters of lacrimal gland function, was measured by an observer blinded to the experimental groups before exposure to ozone (baseline), and after 1 and 2 weeks of exposure. Tear volume was measured using the phenol red thread test, as described elsewhere.[29] Briefly, after slightly lowering the lower eyelid, the threads were held with jeweler forceps and placed in the lateral cantus of the conjunctival fornix for 30 seconds. The tear distance (in millimeters) was read under a microscope (model E800; Nikon, Melville, NY, USA) using the hemocytometer scale. The final reported length was an average of three separate measurements.

### Conjunctival epithelial cells and stimulation of cytokines

A human conjunctival cell line (Wong Kilbourne derivative of Chang conjunctiva, clone 1-5c-4, CCL–20.2; American Type Culture Collection, Manassas, VA, USA) was cultured under standard conditions (humidified atmosphere of 5% CO_2_ at 37°C) in Medium 199 supplemented with 10% fetal calf serum and 1% ampicillin/streptomycin. Confluent cultures were removed by incubation in 0.25% trypsin.

IL-1α (10 ng/mL; R&D Systems, Minneapolis, MN, USA) was added to 80%–90% confluent conjunctival epithelial cells for 24 hours to enhance conjunctival inflammation, mimicking the *in vivo* model of EAC. Conjunctival epithelial cells without or with IL-1α (10 ng/mL) pretreatment were exposed to 0.5 ppm and 2.0 ppm of ozone for 1 hour in an exposure chamber. An environmental exposure system that allowed for simultaneous exposure to ozone to the cells was used. We monitored and adjusted the volume of the medium covering the surface of the culture plate.

### Cell viability assays

Cell viability was evaluated using a colorimetric method based on highly sensitive water-soluble tetrazolium salts (WST; CellVia-1000T; Abfrontier, Seoul, Korea), which are reduced by living cells to yield purple formazan crystals. Briefly, conjunctival epithelial cells without and with IL-1α pretreatment were incubated with ozone (0.5 or 2.0 ppm) for 1 and 2 hours. The cells were then washed and treated with WST. The plate was incubated in the dark at 37°C and in a humidified atmosphere containing 5% CO_2_ for 1 hour. After incubation, the culture supernatant was removed using a micropipette. The plate was mixed horizontally for 1 minute and the optical density was measured at 540 nm using a microplate reader. Cell survival was expressed as the percentage of absorbance relative to that of the untreated cells. The results are representative of at least three independent experiments.

### Western blot analysis

Cells were lysed with RIPA buffer (Biosesang, Inc., Seoul, Korea) containing 5 mM ethylenediaminetetraacetic acid, 1 mM phenylmethylsulfonyl fluoride, 1 mM sodium orthovanadate, 1 μg/mL pepstatin, and 10 μg/mL leupeptin for 20 minutes at 4°C, scraped with a cell scraper, and centrifuged at 15,000 × *g* for 15 minutes at 4°C. The cell lysates were boiled in Laemmli sample buffer (Bio-Rad, Hercules, CA, USA) for 5 minutes. Proteins were separated by sodium dodecyl sulfate-polyacrylamide gel electrophoresis (Bio-Rad) on 12% gels and transferred to polyvinylidene difluoride membranes (Millipore). The membranes were blocked overnight at 4°C in 5% bovine serum albumin or 5% nonfat dry milk in a buffer containing 10 mM Tris-HCl (pH 8.0) (Sigma-Aldrich), 150 mM NaCl, and 0.05% Tween-20 (Sigma-Aldrich), and then incubated overnight with the primary antibodies for manganese superoxide dismutase (Mn-SOD; Stressgen, Victoria, BC, Canada), catalase (Abcam, Cambridge, UK), thioredoxin reductase-1 (TRXr-1; Santa Cruz Biotechnology, Santa Cruz, CA, USA), heme-oxygenase-1 (HO-1; Santa Cruz Biotechnology), total OXPHOS Complexes Detection Kit (Mitosciences Inc., Eugene, OR, USA), peroxisome proliferator-activated receptor-γ coactivator-1α (PGC-1α; Novus Biologicals, LLC, Littleton, CO, USA), voltage-dependent anion channel (VDAC; Cell Signaling Technology, Beverly, MA, USA), mammalian target of rapamycin (mTOR), phospho-mTOR (p-mTOR, Cell Signaling Technology), Akt, p-Akt (Cell Signaling Technology), Lamin A/C (Cell Signaling Technology), or β-actin (Santa Cruz Biotechnology), followed by incubation with peroxidase-labeled anti-mouse IgG secondary antibody (KPL Laboratories, Gaithersburg, MD, USA) or peroxidase-labeled anti-rabbit IgG secondary antibody (KPL Laboratories). β-actin served as a loading control. Immunoreactive bands were visualized using an enhanced chemiluminescence Western blotting kit (Amersham Pharmacia Biotech, Piscataway, NJ, USA) according to the manufacturer's instructions. The results are representative of at least three independent experiments.

### Reverse transcription polymerase chain reaction

Total RNA was isolated from monolayers of cultured conjunctival cells using TRIzol reagent (Invitrogen, San Diego, CA, USA). cDNA was synthesized from 1 μg of total RNA using cDNA EcoDry Premix (Clontech Laboratories, Inc., Mountain View, CA, USA) according to the manufacturer’s protocol. One microgram of cDNA was subsequently used for reverse transcription polymerase chain reaction (RT-PCR). The housekeeping gene, β-actin, was used as an internal control. Targets were amplified using individually optimized thermocycling conditions. The following primer sets were used: IL-6 forward, 5’-CCTTCTCCACAAGCGCCTTC-3’ and reverse, 5’-GGCAAGTCTCCTCATTGAATC-3’; IL-8 forward, 5’-ATGACTTCCAAGCTGGCCGTGGCT-3’ and reverse, 5’-TCTCAGCCCTCTTCAAAAACTTCTC-3’; IL-17 forward, 5’-CAAAATTCCAAGTTCTCGATTTCACA-3’ and reverse, 5’-TGGGCTGAACTTTTCTCATACTTAAA-3’; interferon (IFN)-γ forward, 5’-GCTTTATCTCAGGGGCCAAC-3’ and reverse, 5’-TGGCTCAGATTGCAGGCATA-3’; TNF-α forward, 5’-GTCAACCTCCTCTCTGCCAT-3’ and reverse, 5’-CCAAAGTAGACCTGCCCAGA-3’; β-actin forward, 5’-GGACTTCGAGCAAGAGATGG-3’ and reverse, 5’-AGCACTGTGTTGGCGTACAG-3’. The ozone-induced expression of IL-6 in conjunctival epithelial cells pretreated with BHA (100 μM/mL) and IL-1α (10 ng/mL) for 24 hours was assessed by RT-PCR. Amplification of β-actin was used as an endogenous reference for determination of the integrity of mRNA in each sample. The amplification products were separated by electrophoresis on 1.7% agarose DNA gels and visualized by ethidium bromide staining. The results are representative of at least three independent experiments.

### MitoTracker and cytochrome c staining

Mitochondria were detected using MitoTracker stock solution (Invitrogen). Before and after exposure to 2.0 ppm ozone for 1 hour, cells without and with IL-1α pretreatment were fixed in Medium 199 containing 4% formaldehyde for 1 hour. After incubating with PBS containing 0.2% Triton X-100 for 10 minutes, the cells were washed with PBS containing 0.02% Tween-20 and treated with 1% bovine serum albumin for 5 minutes. The cells were then stained with 1 mmol/L MitoTracker Red 580 (Molecular Probes, Inc., Eugene, OR, USA) for 30 minutes. After staining, the cells were washed three times with serum-free Medium 199. For cytochrome c staining, the cells were incubated with goat anti-cytochrome c antibody (Abcam) to a final working concentration of 1:650 in PBS containing 3% bovine serum albumin. After several washes in buffer, Alexa Fluor 594 donkey anti-rabbit IgG (Invitrogen) was applied as a secondary antibody. Images of stained cells were acquired using a confocal microscope (TCS-SP5; Leica Microsystems Inc., Bannockburn, IL, USA), and their morphology and staining intensity were analyzed using Scion Image software (Scion Corp., Frederick, MD, USA).

### Statistical analysis

Data are reported as the mean ± standard error of the mean. Statistical analysis in each independent experiment was performed using an unpaired *t*-test. A paired *t*-test was used for comparison of data before and after exposure to ozone. One-way analysis of variance and Bonferroni's post hoc comparison test were used to compare the means of multiple groups. Statistical analyses were performed using GraphPad PRISM software (GraphPad Software, Inc., La Jolla, CA, USA). Differences were considered to be statistically significant at *p*-values of < 0.05.

### Ethics statement

All experiments involving animal subjects were conducted in strict accordance and adherence to relevant national and international guidelines regarding animal handling as mandated by the IACUC of the Yonsei University Health System, Seoul, Korea (ethics approval number 2014–0107).

## Results

### Effect of ozone on clinical outcomes in an experimental model of allergy

**Fig 1:**
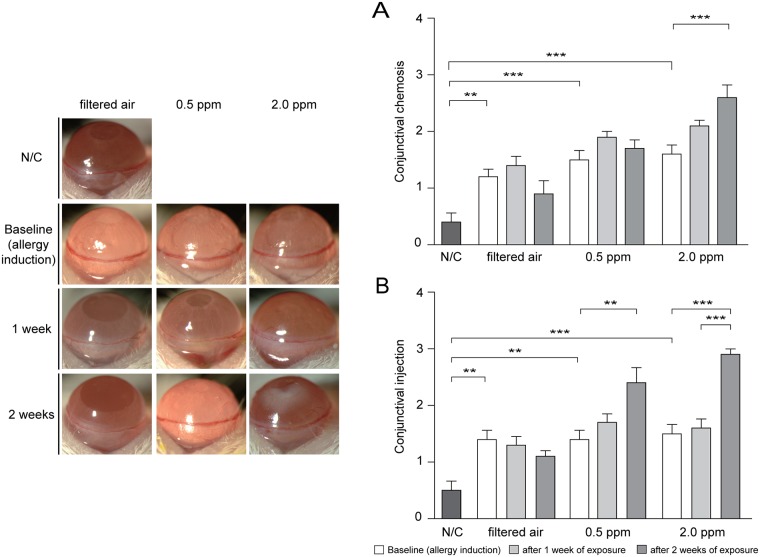
Changes in conjunctival chemosis and conjunctival injection after exposure to ozone in a mouse model of experimental allergic conjunctivitis. (A) Conjunctival chemosis. (B) Conjunctival injection. N/C, negative control; 1 week, after 1 week of exposure; 2 weeks, after 2 weeks of exposure. Error bars represent the standard error of the mean (**p < 0.01, ***p < 0.001).

Using a mouse model of EAC, we investigated the effects of two different concentrations of ozone (0.5 ppm and 2.0 ppm) on conjunctival chemosis, conjunctival injection, and corneal and conjunctival fluorescein staining scores. Baseline (OVA-induced allergic conjunctivitis, but prior to exposure to ozone) conjunctival chemosis and conjunctival injection scores in groups B, C, and D were significantly higher than in group A (Fig 1). In the presence of 2.0 ppm ozone (group D), the conjunctival chemosis scores after 2 weeks of exposure were significantly higher than those at baseline (*p* < 0.001, Fig 1A). There was a significant increase in conjunctival injection scores after 2 weeks of exposure to 0.5 ppm ozone (*p* < 0.01, Fig 1B). In the presence of 2.0 ppm ozone (group D), conjunctival injection scores after 2 weeks of exposure were significantly higher than those at baseline and after 1 week of exposure (*p* < 0.001, Fig 1B). Moreover, there were significant differences in conjunctival chemosis scores between groups B, C, and D after 1 and 2 weeks of exposure (*p* = 0.002 for 1 week and *p* < 0.001 for 2 weeks), and the scores demonstrated an ozone dose-dependent pattern. The conjunctival injection scores were significantly different between groups B, C, and D after 2 weeks of exposure (*p* < 0.001), and again the scores demonstrated an ozone-dose dependent pattern.

**Fig 2:**
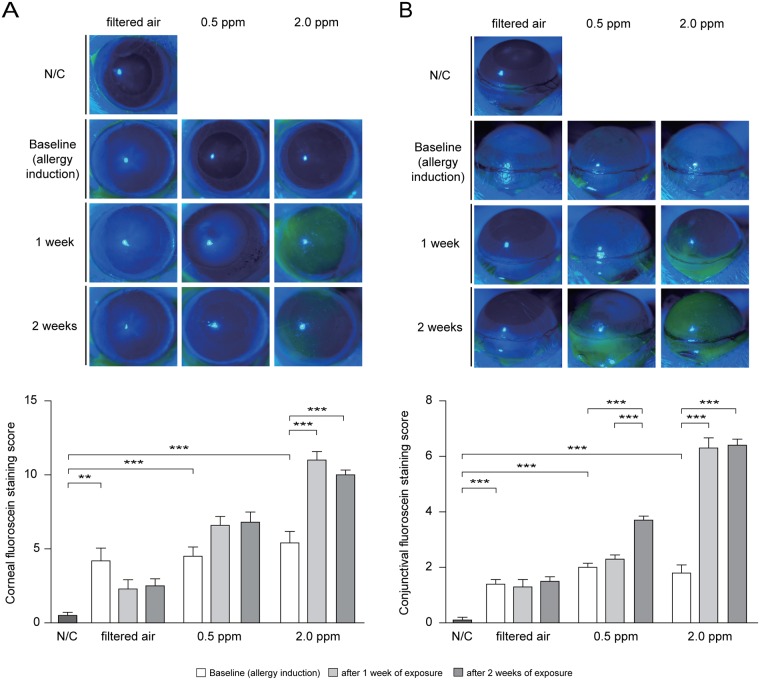
Changes in corneal and conjunctival fluorescein staining scores after exposure to ozone in a mouse model of experimental allergic conjunctivitis. (A) Corneal fluorescein staining scores. (B) Conjunctival fluorescein staining scores. N/C, negative control; 1 week, after 1 week of exposure; 2 weeks, after 2 weeks of exposure. Error bars represent the standard error of the mean (**p < 0.01, ***p < 0.001).

Baseline corneal and conjunctival fluorescein staining scores for groups B, C, and D were significantly higher than in group A (Fig 2A and 2B). In the presence of 2.0 ppm ozone (group D), corneal fluorescein staining scores after 1 and 2 weeks of exposure were significantly higher than those at baseline (*p* < 0.001, Fig 2A). There were no significant differences in corneal fluorescein staining scores during the course of the study in either group B or group C (Fig 2A). In the presence of 0.5 ppm ozone (group C), conjunctival fluorescein staining scores after 2 weeks of exposure were significantly higher than those at baseline and after 1 week of exposure (*p* < 0.001, Fig 2B). In the presence of 2.0 ppm ozone (group D), conjunctival fluorescein staining scores were significantly higher after 1 and 2 weeks of exposure than those at baseline (*p* < 0.001, Fig 2B). Moreover, there were significant differences in the corneal and conjunctival fluorescein staining scores between groups B, C, and D after 1 and 2 weeks of exposure (all *p* < 0.001). The scores again demonstrated an ozone dose-dependent pattern.

### Effect of ozone on concentrations of inflammatory cytokines in tears and aqueous tear production in an experimental model of allergy

**Fig 3:**
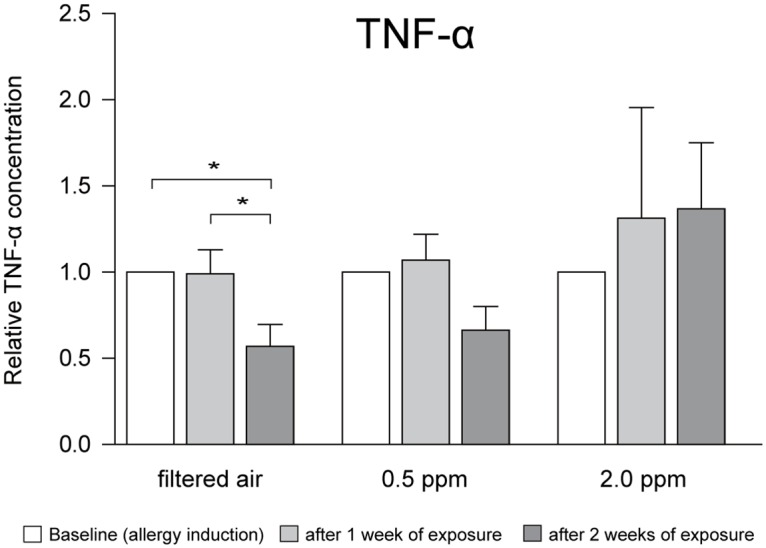
Changes in tumor necrosis factor- α concentrations in tears after exposure to ozone in a mouse model of experimental allergic conjunctivitis. **TNF, tumor necrosis factor.** Error bars represent the standard error of the mean (*p < 0.05).

Using a mouse model of EAC, we next investigated whether ozone affected inflammatory cytokine concentrations in tears. TNF-α levels in a mouse model of EAC with exposure to filtered room air decreased after 2 weeks when compared with the levels at baseline and after 1 week of exposure. No significant decrease in TNF-α levels was noted on exposure to 0.5 ppm ozone (group C). On exposure to 2.0 ppm ozone (group D), TNF-α levels tended to increase in a time-dependent manner, albeit not significantly (Fig 3). IL-1β, IL-6, and IL-17 levels showed no significant changes in response to exposure to ozone (data not shown).

**Fig 4:**
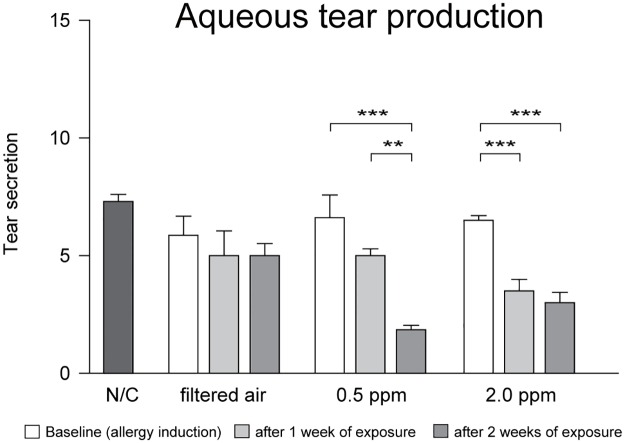
Changes in aqueous tear production after exposure to ozone in a mouse model of experimental allergic conjunctivitis. N/C, negative control. Error bars represent the standard error of the mean (**p < 0.01, ***p < 0.001).

The total tear volume in groups B, C, and D at baseline was not significantly different from that in group A (Fig 4). In the presence of 0.5 ppm ozone (group C), the total tear volume after 2 weeks of exposure was significantly lower than that at baseline and after 1 week of exposure (*p* < 0.001 and *p* < 0.01, respectively). In the presence of 2.0 ppm ozone (group D), total tear volume after 1 and 2 weeks of exposure was significantly lower than that at baseline (*p* < 0.001). In mice with OVA-induced allergic conjunctivitis, total tear volume was shown to be affected by exposure to ozone.

### Effect of IL-1α and ozone on inflammatory cytokines in cultured conjunctival epithelial cells

**Fig 5:**
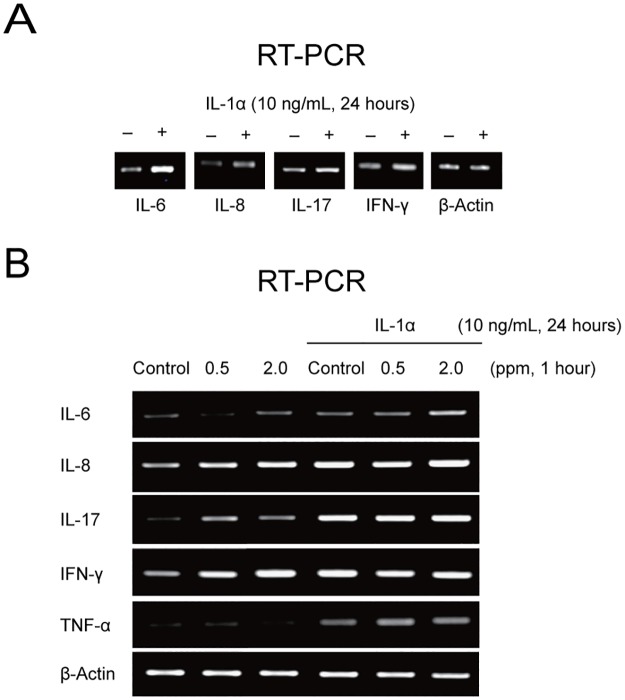
Changes in expression of inflammatory cytokines after treatment with IL-1α in conjunctival epithelial cells and after exposure to ozone in IL-1α-pretreated conjunctival epithelial cells. (A) Treatment with IL-1α. (B) Ozone exposure in IL-1α-pretreated conjunctival epithelial cells. IL, interleukin; IFN, interferon; TNF, tumor necrosis factor.

Using RT-PCR, we investigated whether treatment with IL-1α induced changes in inflammatory cytokines (IL-6, IL-8, IL-17, and IFN-γ) in cultured human conjunctival epithelial cells. Treatment with IL-1α (10 ng/mL) for 24 hours increased the expression of IL-6, IL-8, IL-17, and IFN-γ mRNA (Fig 5A). This suggests that IL-1α-pretreated conjunctival epithelial cells could act as a substitute *in vitro* model of the *in vivo* mouse model of OVA-induced allergic conjunctivitis in terms of the response of inflammatory cytokines. We next investigated the effects of exposure to ozone on the expression of inflammatory cytokines (IL-6, IL-8, IL-17, IFN-γ, TNF-α) in cultured human conjunctival epithelial cells without and with IL-1α pretreatment using RT-PCR. Ozone in conjunctival epithelial cells not pretreated with IL-1α induced an increase in IL-6, IL-8, IL-17, and IFN-γ mRNA levels. IL-1α also induced increased expression of IL-6, IL-8, IL-17, IFN-γ, and TNF-α. In the IL-1α-pretreated cells, ozone induced an additional increase in IL-6 and TNF-α mRNA levels (Fig 5B). In the case of IL-6, IL-17, and TNF-α, levels of mRNA expression after exposure to ozone 0.5 ppm or 2.0 ppm in IL-1α-pretreated cultured human conjunctival epithelial cells were higher than those in cells not pretreated with IL-1α (Fig 5B).

### Effect of ozone on viability of conjunctival epithelial cells with and without IL -1α pretreatment

We next investigated whether ozone induced changes in the viability of cultured human conjunctival epithelial cells without or with IL-1α pretreatment. After incubation with exposure to ozone 0.5 or 2.0 ppm for 1 and 2 hours, the cells showed no changes in viability (data not shown).

### Effect of ozone on expression of antioxidant enzyme and oxidative stress markers in cultured conjunctival epithelial cells

**Fig 6:**
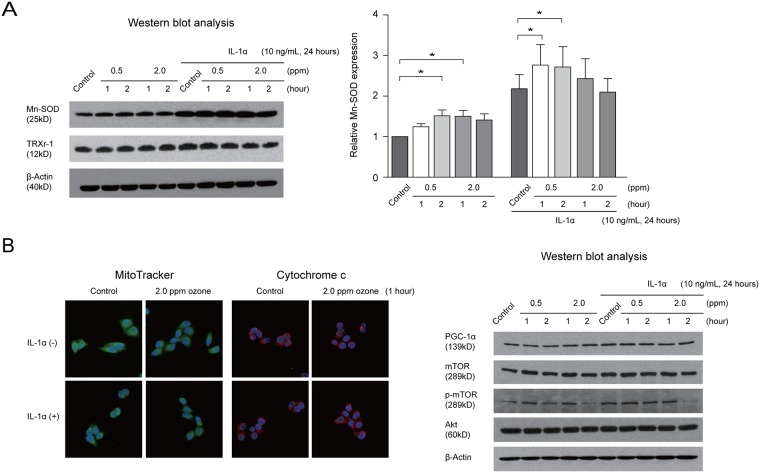
Changes in expression of antioxidant enzyme, oxidative stress marker, mitochondrial activity, and proteins involved in mitochondrial function after exposure to ozone in IL-1α-pretreated conjunctival epithelial cells. (A) Antioxidant enzyme and oxidative stress marker. (B) Mitochondrial activity and proteins involved in mitochondrial function. IL, interleukin; Mn-SOD, manganese superoxide dismutase; TRXr-1, thioredoxin reductase-1; PGC-1α, peroxisome proliferator-activated receptor-γ coactivator-1α; mTOR, mammalian target of rapamycin; p-mTOR, phospho-mammalian target of rapamycin. Error bars represent the standard error of the mean (*p < 0.05).

We next investigated whether exposure to ozone induced changes in the expression of antioxidant enzymes (Mn-SOD, catalase) and stress-responsive proteins (TRXr-1, HO-1) in cultured human conjunctival epithelial cells without and with IL-1α pretreatment using Western blot analysis. Levels of oxidative stress markers did not change in a statistically significant manner in cultured human conjunctival epithelial cells without and with IL-1α pretreatment after incubation with ozone 0.5 ppm or 2.0 ppm (all *p* > 0.05; data not shown). Expression of Mn-SOD in cultured human conjunctival epithelial cells was significantly increased after pretreatment with IL-1α, and was induced further by exposure to ozone (Fig 6A).

### Effect of ozone on mitochondrial activity and expression of mitochondrial enzymes and proteins related to mitochondrial function

The morphology and intensity of MitoTracker and cytochrome c staining in human conjunctival epithelial cells did not change when the cells were or were not pretreated with IL-1α. Ozone did not induce any change in morphology and staining intensity in cultured human conjunctival epithelial cells with or without IL-1α pretreatment (Fig 6B). To determine whether ozone induced changes in expression of mitochondrial proteins in cultured human conjunctival epithelial cells, levels of randomly selected subunits of complexes I–V in the respiratory chain were measured by Western blot analysis. Levels of these complexes were not significantly changed (all *p* > 0.05; data not shown). Expression of p-mTOR after exposure to ozone 2.0 ppm for 2 hours was significantly decreased when compared with the control in cultured human conjunctival epithelial cells pretreated with IL-1α (*p* < 0.05; Fig 6B).

### Effect of ozone on expression of inflammatory cytokines in butylated hydroxyanisole-pretreated cultured conjunctival epithelial cells

**Fig 7:**
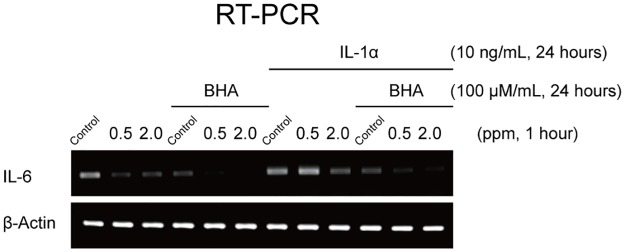
Changes in expression of IL-6 after exposure to ozone in IL-1α and butylated hydroxyanisole-pretreated conjunctival epithelial cells. IL, interleukin; BHA, butylated hydroxyanisole.

We investigated the effects of exposure to ozone on expression of IL-6 in BHA-pretreated cultured conjunctival epithelial cells using RT-PCR. The cells were co-treated with BHA (100 μM/mL) and IL-1α for 24 hours and then exposed to ozone 0.5 and 2.0 ppm for 1 hour. Treatment with BHA attenuated ozone-induced increases in IL-6 expression in cultured conjunctival epithelial cells upon treatment with IL-1α (Fig 7).

## Discussion

The ocular surface is easily damaged by air pollutants such as ground ozone because it is directly exposed to the external environment. Conjunctival epithelial cells are one of the first types of cells to come in contact with ozone, and are major mediators of ozone-induced reactions on the ocular surface. Moreover, conjunctival epithelial cells have a crucial role in the development of conjunctival allergic reactions in response to provocation with specific allergens.

Our group recently demonstrated the effect of ozone on the ocular surface using *in vitro* and *in vivo* models. In line with studies that have evaluated the deleterious impact of exposure to ozone on allergic asthma, [17–20] in this study we investigated the effect of exposure to ozone on allergic conjunctivitis. We assessed the ocular effects of ozone in a mouse model of EAC by evaluating multiple ocular surface parameters, including conjunctival chemosis, conjunctival injection, corneal and conjunctival fluorescein staining scores, concentrations of inflammatory cytokines in tears, and aqueous tear production. Using an *in vitro* model consisting of IL-1α-pretreated conjunctival epithelial cells, we also investigated whether exposure to ozone induces an inflammatory response and alters oxidative stress states and mitochondrial function.

Our results demonstrate that exposure to ozone exacerbates deterioration of the ocular surface and amplifies the inflammatory state already induced by an allergic reaction, as evidenced by an increase in conjunctival chemosis, conjunctival injection, and corneal and conjunctival fluorescein staining scores in a mouse model of EAC. Moreover, we found that conjunctival fluorescein staining scores and conjunctival injection were affected by exposure to lower dose ozone after induction of allergic conjunctivitis. Previously, we reported that exposure to ozone did not affect aqueous tear production.[3] However, in an *in vivo* model of EAC, exposure to ozone induced a significant decrease in tear production. Considering that ozone irreversibly damaged corneal integrity and conjunctival goblet cell density *in vivo*, we concluded that exposure to ozone and the allergic reaction in conjunctival epithelial cells had an additional adverse effect on the ocular surface that led to decreased tear production.[3]

To validate our *in vitro* model, we investigated whether treatment with IL-1α increases the production of inflammatory cytokines (IL-6, IL-8, IL-17, and IFN-γ) in cultured human conjunctival epithelial cells. The IL-1 family is involved in the pathogenesis of several acute and chronic inflammatory diseases. Further, IL-1 has been reported to induce cellular responses that cause airway hyper-responsiveness following exposure to ozone.[12,30] The IL-1 pathway was recently reported to be associated with airway inflammation in response to exposure to ozone in individuals with allergic asthma.[18] IL-1α in particular has emerged as a major damage-associated molecular pattern and an inducer of inflammation in a variety of conditions.[31–35] Our results show that administering IL-1α (10 ng/mL) for 24 hours increased the expression of IL-6, IL-8, IL-17, and IFN-γ. Thus, cells treated with IL-1α could act as a substitute for the *in vivo* mouse model of EAC in terms of the inflammatory response, even though they do not exhibit the pathology of allergic conjunctivitis.

We demonstrated that expression of IL-6, IL-8, IL-17, and IFN-γ was increased after exposure to ozone in cultured human conjunctival epithelial cells not pretreated with IL-1α, which is in line with our previous results.[3] We also found that exposure to ozone further increased the expression of IL-6 and TNF-α in IL-1α-pretreated cultured human conjunctival epithelial cells. Further, expression of IL-6, IL-17, and TNF-α in IL-1α-pretreated cultured human conjunctival epithelial cells after exposure to ozone was higher than that after exposure to ozone in cells not pretreated with IL-1α. Thus, we speculate that the increased expression of IL-6 and TNF-α after exposure to ozone can trigger an inflammatory reaction and have a detrimental effect on the ocular surface. Our hypothesis is supported by the results for multiple ocular surface parameters, including conjunctival chemosis, conjunctival injection, corneal and conjunctival fluorescein staining scores, concentrations of inflammatory cytokines in tears, and aqueous tear production in our *in vivo* model. Concentrations of TNF-α in our mouse model of EAC decreased after 2 weeks of exposure to filtered room air when compared with baseline and 1 week of exposure to filtered room air. However, there was no significant decrease in TNF-α levels in the groups exposed to ozone 0.5 ppm or 2.0 ppm. Moreover, TNF-α levels in the group exposed to ozone 2.0 ppm showed a tendency to increase. Even taking into account the discrepant findings between our *in vitro* model and *in vivo* mouse model of EAC, the ozone-induced changes in TNF-α levels in the mouse model of EAC are notable.

Pretreatment with IL-1α increased the expression of Mn-SOD, which was further increased by subsequent exposure to ozone. IL-1α has been reported to upregulate the expression of Mn-SOD, a mitochondrial enzyme involved in the detoxification of ROS in several cell lines.[36–38] Ozone exposure did not increase the expression of stress-responsive proteins (HO-1, TRXr-1). Additionally, the free radical scavenger BHA attenuated the ozone-induced increases in IL-6 expression in our *in vitro* model. These results suggest that exposure to ozone may have a role in the exacerbation of inflammation in IL-1α-treated conjunctival epithelial cells, while simultaneously affecting the oxidative stress system. Further research investigating the defense or antioxidant mechanisms associated with exposure to ozone without or with the existence of inflammation should be performed *in vitro* and *in vivo*. In a recent study evaluating the effects of exposure to ozone on the lipid component of tear film, the authors reported no signs of ozonolysis products in the tear fluid; this finding was most attributable to the antioxidant mechanisms in tear fluid and detoxification enzymes present in the cornea.[39,40]

To evaluate the effect of ozone on the mitochondrion, we assessed mitochondrial activity using MitoTracker and cytochrome c staining. There were no significant differences in morphology and staining intensity before and after treatment with IL-1α. Moreover, even after exposure to ozone, no significant change in morphology and staining intensity was noted. In line with these results, cellular viability was maintained after exposure to ozone in cultured human conjunctival epithelial cells without and with IL-1α pretreatment. These results are consistent with those of our previous study showing that ozone did not induce apoptosis in cultured conjunctival epithelial cells.[3]

The membrane-bound mitochondrial complexes I–V establish a proton gradient across the mitochondrial membrane, thus producing a thermodynamic state with the potential to do work. No significant changes in the expression of complexes I–V were noted, indicating that the role of the mitochondria in generating energy and performing redox reactions is maintained in conjunctival cells after exposure to ozone.

mTOR is a large serine/threonine protein kinase that forms multiple subunit complexes with numerous protein partners. These protein complexes have distinct biological functions, including mediating metabolism during aging and regulating mitochondrial oxygen consumption and oxidative capacity via the Akt/mTOR pathway.[41] Transcriptional complexes that contain PGC-1α control mitochondrial oxidative function to maintain energy homeostasis. Cytochrome c passes through the mitochondrial permeability transition pore, which is made up of the VDAC in the outer membrane, the adenine nucleotide translocator in the inner membrane, and several auxiliary proteins that include the Bcl-2 family. Thus, VDAC is an important molecule in mitochondria-mediated apoptosis, and may play a role in regulating release of cytochrome c.[42] With the exception of p-mTOR, expression of these proteins was maintained after exposure to ozone in cultured human conjunctival epithelial cells without and with IL-1α pretreatment.

The main methodologic limitation of this study was the inevitable discrepancy between our *in vitro* model and the *in vivo* mouse model of EAC. As far as we could ascertain, there are no *in vitro* models for allergic conjunctivitis available at present. In the present study, nonetheless, we produced an *in vitro* model and used it for our investigations, with the caveat that it cannot demonstrate the exact pathognomonic signs of allergic conjunctivitis.

Nevertheless, our results are valuable in terms of elucidating the cellular events underlying ozone-induced changes associated with inflammation on the ocular surface, and laying the foundation for further translational research involving ambient ozone-exposed human subjects with allergic conjunctivitis.

**Fig 8:**
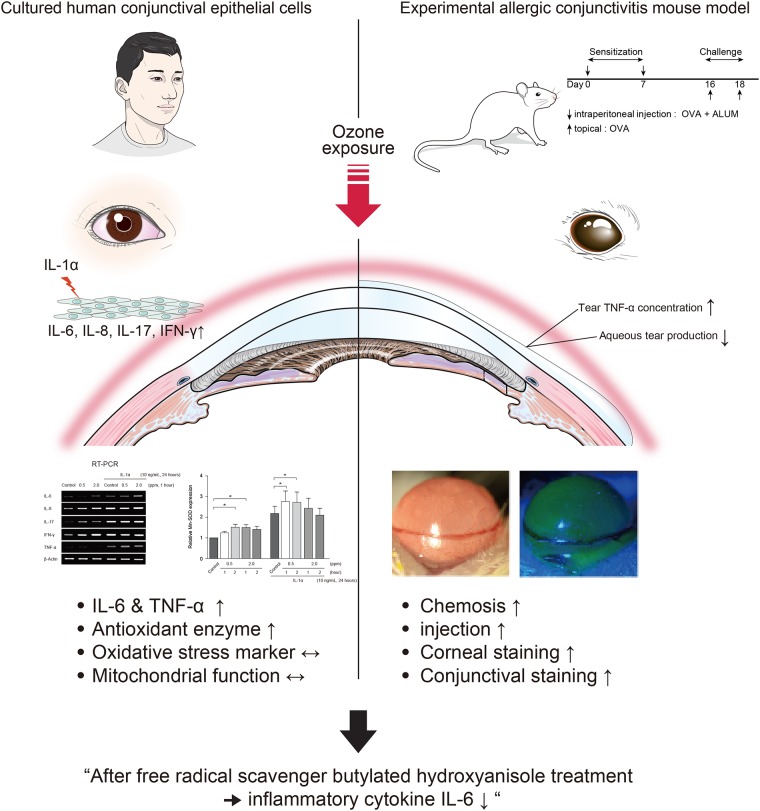
Summary of the current study.

In conclusion, we have demonstrated that exposure to atmospheric ozone exacerbates damage to the ocular surface and decreases tear production in an *in vivo* model of EAC. Further, on the basis of our results in an *in vitro* model, we conclude that exposure to ozone does not cause mitochondrial function to deteriorate via increased expression of antioxidant enzymes, but may enhance the inflammatory response without altering cellular viability (Fig 8).

## Supporting Information

S1 File Clinical results demonstrating the effects of exposure to ozone on the ocular surface in a mouse model of experimental allergic conjunctivitis. (XLSX)
