# Correction: Effects of Exposure to Ozone on the Ocular Surface in an Experimental Model of Allergic Conjunctivitis

**DOI:** 10.1371/journal.pone.0173328

**Published:** 2017-02-28

**Authors:** Hun Lee, Eung Kweon Kim, Hee Young Kim, Tae-im Kim

The following information is missing from the Funding section: This work was supported by a grant of the Korean Health Technology R & D Project, Ministry of Health & Welfare, Republic of Korea (HI14C2044).
